# Development and Evaluation of a PCV‐Dependent Visual Hemolysis Color Scale for Packed Red Blood Cell Products

**DOI:** 10.1111/vec.70017

**Published:** 2025-08-29

**Authors:** Hyein Jung, K. Jane Wardrop, Sabrina N. Hoehne, Linda G. Martin, Jillian M. Haines, Trey L. DeJong, Elizabeth B. Davidow

**Affiliations:** ^1^ Department of Veterinary Clinical Sciences, College of Veterinary Medicine Washington State University Pullman Washington USA; ^2^ Department of Mathematics and Statistics, College of Arts and Sciences Washington State University Pullman Washington USA

**Keywords:** access to care, blood product, blood transfusion/standards, hemoglobin, transfusion reaction

## Abstract

**Objective:**

To develop a PCV‐dependent hemolysis color scale and evaluate its accuracy in predicting supernatant hemoglobin concentration in packed red blood cell (pRBC) products, helping to determine transfusion safety.

**Design:**

Prospective experimental study.

**Setting:**

University veterinary teaching hospital.

**Interventions:**

Serial dilutions of a pRBC unit were performed to create a range of hemolyzed supernatant samples. A commercial graphics program was used to allocate computerized color to each hemolyzed sample, constructing a color scale. Study participants were then asked to use the color scale to estimate the hemoglobin concentration of a provided supernatant sample. The color estimation data were analyzed by the authors to determine whether to transfuse blood, as pRBC should not be transfused if the product's percentage hemolysis is >1%.

**Measurements and Main Results:**

Visual inspection with the color scale was evaluated with seven supernatant test samples containing different free hemoglobin concentrations (0.3–8.0 g/L). The percentage of correct color estimation overall was 61.9%. The percentage of correct decisions to transfuse the blood product overall would have been 93.7%. All incorrect estimations were one color range (approximately 1.0 g/L difference between the ranges) off from the correct estimation for all samples.

**Conclusions:**

The color scale aided the visual assessment of hemolysis. However, visual inspection with the color scale can still be inaccurate near the corresponding cutoff color of <1% hemolysis in each PCV range. If the supernatant color of the unit is estimated to be one color range off from the cutoff color, the cell‐free hemoglobin concentration of the unit should be measured, and percentage hemolysis should be calculated to determine if it meets the <1% hemolysis standard.

**Abbreviations**
FDA: Food and Drug Administration
pRBC: packed red blood cell

## 1 Introduction

The administration of hemolyzed blood products to transfusion recipients can produce clinically important complications. Hemoglobin, a tetramer composed of heme and globin, is contained within RBCs and plays an important role in gas transport and delivery by binding and releasing oxygen molecules. It is also intrinsically toxic due to its production of reactive oxygen species, nitric oxide scavenging, and a tendency for dimerization if exteriorized from the RBC. Hemoglobin's toxicity is minimized by compartmentalization (i.e., hemoglobin being encapsulated within the RBC), an intracellular antioxidant system within the RBC, and plasma protein buffers (e.g., haptoglobin and hemopexin) that bind cell‐free hemoglobin and heme, leading to the degradation of both. In pathologic conditions such as intravascular hemolysis or the administration of a hemolyzed RBC blood product, large amounts of hemoglobin and heme are released into the extracellular space, and scavenging proteins are overwhelmed. Such hemoglobinemia leads to the depletion of nitric oxide and oxidative injury by cell‐free hemoglobin and heme, which may lead to vasoconstriction, gastrointestinal dystonia and pain, platelet activation, endothelial dysfunction, and acute kidney injury [1, 2, 3, 4].

Hemolysis of RBC‐based blood products is also a marker of RBC storage lesions, indicating that biochemical, biomechanical, and immunologic changes have developed as a result of altered metabolism and scavenging systems in the face of an ex vivo environment [5]. These changes include decreases in ATP, accumulation of ammonia, changes in RBC shape, and accumulation of microparticles (small anucleate phospholipid‐rich vesicles) [5]. Previous research in people has shown that the transfusion of older RBC units containing storage lesions is associated with an increased risk of death, multiple organ dysfunction syndrome, pneumonia, renal dysfunction, sepsis, infection, deep vein thrombosis, ICU length of stay, and postoperative complications [6, 7, 8]. Veterinary studies have also indicated that hemolysis can develop in blood units, which may increase patient risk [9, 10, 11].

When new RBC blood product storage solutions are developed, manufacturers are required to demonstrate to the US Food and Drug Administration (FDA) that at least 75% of the transfused RBCs remain in circulation for 24 h after the transfusion. This is done by performing radiolabeling studies in healthy volunteers and showing that the percentage hemolysis of the RBC blood products is <1% at the end of storage [12]. The percentage hemolysis is calculated as (100 − PCV) × free plasma hemoglobin/total hemoglobin [13]. The regulation of percentage hemolysis follows the “95/95 rule,” by which the manufacturer of the blood storage bag and solution should prove that 95% of the units meet the standard with 95% statistical certainty [24]. Although there is no FDA requirement regarding the degree of hemolysis in canine or feline RBC units, the recent Association of Veterinary Hematology and Transfusion Medicine Transfusion Reaction Small Animal Consensus Statement guidelines recommend that canine and feline RBC units are checked for hemolysis before administration and are not used if the hemolysis percentage is ≥1% [14]. The routine storage time for canine RBCs with various additive solutions has been determined in part based on this 1% standard of hemolysis [15, 16, 17, 18, 19].

The only method to accurately measure free hemoglobin in blood bags is to use a hemoglobin meter, which many veterinary hospitals do not have. Although visual inspection of blood units for hemolysis is used in people just before administration to a patient, visual inspection has been shown to be deceptive and inaccurate [20]. A previous veterinary study has also reported the inaccuracy of visual inspection, some of which may be because the standard is based on a simple percentage, and thus, the acceptable free hemoglobin will vary based on the total hemoglobin in the unit [13]. The total hemoglobin contained within a unit is anticipated to correlate with the unit's PCV. Thus, packed red blood cell (pRBC) units that have a higher PCV and therefore higher total hemoglobin concentration should have a higher absolute acceptable free hemoglobin level and a darker supernatant color than less concentrated units.

Currently available color scales in human medicine do not consider PCV as a factor that changes the percentage of acceptable hemolysis for a given blood unit, and there is no hemolysis color scale available in veterinary medicine. The objective of the current study was to develop a PCV‐dependent hemolysis color scale for pRBC products and to evaluate whether the accuracy of visual inspection could be improved by using the color scale.

## 2 Materials and Methods

### 2.1 Creation of the Color Scale

**FIGURE 1:**
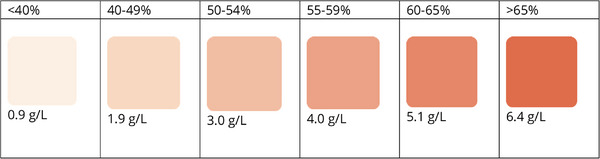
Color scale “cutoff” colors and associated hemoglobin values for different PCV ranges. The scale colors were matched to PCV ranges to become “cutoff” colors (i.e., the highest color and associated cell‐free hemoglobin concentration that could be used with a given unit PCV range to provide <1% hemolysis).

An expired unit of canine pRBCs^a^, produced by Washington State University Veterinary Teaching Hospital Blood Bank, was used to obtain hemolyzed supernatant. The pRBC units consisted of RBCs, a minimal amount of trapped plasma, anticoagulant solution^a^, and a saline dextrose additive solution^a^. The blood unit (∼100 mL) was centrifuged to separate the supernatant and cells. Approximately 10 mL of supernatant was retrieved. A point‐of‐care hemoglobin meter^b^ was used according to the manufacturer's protocol to measure low values of free hemoglobin in the supernatant. Free hemoglobin was initially measured at a value too high to read. An aliquot of supernatant (0.05 mL) was then diluted with an equal volume of 0.9% sodium chloride^c^, and the diluted supernatant was measured for cell‐free hemoglobin at 15.4 g/L. Sodium chloride (0.9%) was used for the serial dilution of the hemolyzed supernatant and was chosen as the diluent solution because it was considered most similar to the supernatant in the pRBC unit. Based on this result, it was determined that 9.74 mL of 1:1 diluted supernatant was needed to make 15 mL of 10 g/L diluted supernatant, which would be the base solution for serial dilutions. The final solution was measured at 10.1 g/L. Using this solution, serial dilutions were performed to make six different cell‐free hemoglobin concentrations of 6.5, 5.0, 4.0, 3.0, 2.0, and 1.0 g/L as measured on the hemoglobin meter. Hematocrit tubes were then filled with the supernatant for each dilution, and the tubes were laid on white paper on a flat desk. The brightness of the site was measured at approximately 500 lux (light meter mobile application^d^). Photographs of each hematocrit tube were taken with a cell phone^e^ using the built‐in camera application^f^. The distance between the camera lens and each hematocrit tube was approximately 25 cm. The angle between the surface of the desk and the direction of the camera to each hematocrit tube was approximately 45 degrees. Camera settings of the photos were ISO 64, 24 mm, 0 ev, f 1.78, and 1/120 s. A computerized color was allocated to each hemolyzed supernatant sample using the graphics program^g^. In this fashion, the different cell‐free hemoglobin concentrations were specifically matched to a color shade, and the Hex color code number of that shade was recorded. These six hemoglobin concentrations and their associated colors were chosen to create the color scale because they were readily discernable from one another visually during a pilot study and represented free hemoglobin ranges that would equal <1% hemolysis in typical PCV ranges (∼40% to ∼65%) of pRBC units (Figure 1).

### 2.2 Visual Inspection of Test Samples Using the Color Scale

The current study was considered exempt from the Washington State University Institutional Review Board. Test samples were visually inspected using the generated color scale. Seven test supernatant samples to be used in the visual inspection were made using an expired canine pRBC unit and the same saline dilution method as previously described. The free hemoglobin concentrations of the test supernatant samples were targeted to be in between the free hemoglobin concentrations adjacent to each other on the color scale, not only to evaluate whether the participant would make a correct estimation but also to evaluate the accuracy of color estimation over the color ranges. The final free hemoglobin concentrations of the test supernatant samples were 8.0, 6.0, 4.4, 3.5, 2.4, 1.4, and 0.3 g/L.

Visual inspection was performed the same day as the test supernatant samples were made. Any people working in the hospital on the day of inspection, including faculty, house officers, veterinary technicians/assistants, and veterinary students, were randomly recruited within 2 h of the test sample production. Supernatant‐filled hematocrit tubes were made with each test supernatant solution. During the initial pilot study, it was noted that the supernatant color in the hematocrit tubes changed overnight; therefore, it was decided to conduct the survey on the same day for only 2 h after the production of the test samples. Visual inspection was performed on a working desk in the ICU. The brightness of the site where the test tubes were placed was measured before the survey and adjusted to be approximately 500 lux using a desk lamp. The color scale was printed on cardboard at a local printing shop and was placed on the desk. Participants were provided with one hematocrit tube at a time and were asked to compare the color of the supernatant to the scale and to choose a color range from seven options (Table 1). Each participant read the same seven test samples of different free hemoglobin concentrations (3.5, 0.3, 6.0, 4.4, 8.0, 2.4, and 1.4 g/L), which were randomly chosen at the start of the study day. Upon color evaluation of the test supernatant, the hematocrit tubes remained on the white portion of the printed color scale and the color scale remained on the desk. The participant was told to not read the color if their hand created a shadow on the reading window. Participants were allowed to move the tube on the scale for color comparison. The selected color range for each supernatant sample was recorded for each participant (Table 1).

**TABLE 1:** Color estimation results showing the distribution of 21 participant responses when comparing seven test samples to color scale ranges.

Color scale ranges, hemoglobin (g/L)								
		× ≤ 0.9	0.9 < × ≤ 1.9	1.9 < × ≤ 3.0	3.0 < × ≤ 4.0	4.0 < × ≤ 5.1	5.1 < × ≤ 6.4	× > 6.4
Test samples, hemoglobin (g/L)	0.3 g/L	20	1	0	0	0	0	0
	1.4 g/L	0	16	5	0	0	0	0
	2.4 g/L	0	2	10	9	0	0	0
	3.5 g/L	0	0	1	18	2	0	0
	4.4 g/L	0	0	0	8	11	2	0
	6.0 g/L	0	0	0	0	11	10	0
	8.0 g/L	0	0	0	0	0	15	6

*Note*: The horizontal axis represents seven different color ranges from which each participant chose an answer for color estimation for each test supernatant sample. The vertical axis represents the free hemoglobin concentration of the seven different test samples. This table should be read by row. The number in the square boxes to the right of the free hemoglobin concentration of each sample represents the number of participants who chose the corresponding color range.

### 2.3 Determination of Participant's Color Identification: Correct Versus Incorrect Color Estimation

If the participant chose the correct color range to which the hemoglobin concentration of the test hematocrit tube belonged, it was determined that they made a correct color estimation. If the participant chose the color range for which the hemoglobin concentration was less than the hemoglobin concentration of the test hematocrit tube, it was determined that they made an underestimation. If the participant chose the color range for which the hemoglobin concentration was higher than the hemoglobin concentration of the test hematocrit tube, it was determined that they made an overestimation.

### 2.4 Determination of Participant's Color Identification: Correct Versus Incorrect Transfusion Decision

In order to determine the potential consequences of incorrect color decisions, six different PCV ranges (<40%, 40%–49%, 50%–54%, 55%–59%, 60%–65%, and >65%) were selected for use with the color scale. These PCV ranges represented typical PCV ranges of stored pRBC units. The free hemoglobin value representing <1% hemolysis was calculated for each PCV range using the formula (100 − PCV) × cell‐free hemoglobin/total hemoglobin <1%, where total hemoglobin in grams per liter was approximated to one third of PCV × 10 [13]. Colors on the scale, when matched to a PCV range, became “cutoff” colors (i.e., the highest color and associated cell‐free hemoglobin concentration that could be used appropriately with that PCV range) (Figure 1).

The participant's color decision data were interpreted using the six different PCV ranges and the cutoff color for cell‐free hemoglobin that gave <1% hemolysis. Based on these data, the authors determined whether the participant would have made a potential correct decision on transfusion of the blood unit regarding hemolysis safety. Giving the blood unit with <1% hemolysis or not giving the blood unit with >1% hemolysis was considered a safe transfusion. The participant's color estimation that would have led to a safe transfusion was considered a correct transfusion decision. If the color range they chose was darker than the cutoff color and the correct color range was darker than the cutoff color, they would choose not to use the blood unit, which would be a safe transfusion decision. If the color range they chose was the same as or lighter than the cutoff color and the correct color range was actually the same as or lighter than the cutoff color, they would decide to use the blood unit, which would also be a correct transfusion decision. Thus, participants could choose an incorrect color range but still make a correct relative color estimation to the cutoff color, which would lead to a safe transfusion decision.

### 2.5 Statistical Methods

Descriptive statistics and software^h^‐based statistical analyses were performed to analyze the data. Chi‐square tests were performed to compare participants’ color estimation (proportion of correct vs. underestimated vs. overestimated) for all evaluated hemoglobin concentrations and to determine if associations existed between PCV groups and whether participants made a correct transfusion decision, as determined by the authors. Cramer *V* effect size was calculated to evaluate the significance of association. Effect sizes were labeled in accordance with Cohen's rules of thumb, in which effect size is considered small, medium, or large if it is 0.04–0.13, 0.13–0.22, or greater than 0.22, respectively [21]. Pairwise comparisons between the test samples were performed using a sequential Sidak in logistic regression to evaluate which hemoglobin concentration differed significantly from each outcome. For all statistical analyses, *p* < 0.05 was considered significant.

## 3 Results

Twenty‐one people (three faculty, three house officers, seven veterinary technicians and assistants, eight veterinary students) participated in this study. A total of 147 sample readings were performed. The overall occurrence of correct color estimation across the test samples was 61.9% (91/147). Chi‐square tests of association with Cramer *V* effect sizes showed that the proportions of correct estimation and underestimation were different between the samples and showed large effect sizes (*χ* ^2^(6) = 31.096, *p* < 0.001, Cramer's *V* = 0.460 in correct estimation, and *χ* ^2^(5) = 20.391, *p* = 0.001, Cramer's *V* = 0.583 in underestimation). The data set for overestimation did not meet the requirement for the chi‐square test; therefore, the results are not reported. Participants were more likely to make a correct estimation with the 0.3, 1.4, and 3.5 g/L samples (95.2%, 76.2%, and 85.7%, respectively), and underestimation was more frequently noted in the 4.4, 6.0, and 8.0 g/L samples (38.1%, 52.4%, and 71.4%, respectively). All incorrect estimations of free hemoglobin concentration were one color range (approximately 1 g/L difference between the ranges) off from the correct estimation in all samples (Table 1).

The percentage of overall correct decisions regarding the transfusion of blood product was 93.7%. A chi‐square analysis showed a statistically significant association between PCV groups and a correct transfusion decision (*χ* ^2^(5) = 13.88, *p* = 0.016, Cramer's *V* = 0.126). The effect size from this finding suggests that differences in correct proportion based on PCV group were medium [21]. The percentages of correct transfusion decisions were 99.32%, 95.24%, 93.20%, 93.20%, 91.16%, and 89.80% for PCV ranges of <40%, 40%–49%, 50%–54%, 55%–59%, 60%–65%, and >65%, respectively. The higher the hemoglobin concentration cutoff and the darker the cutoff color, the more likely participants were to make an incorrect transfusion decision. All wrong decisions about the suitability of the blood product for transfusion for each PCV range occurred when the sample supernatant color was within one color range (approximately 1 g/L difference between the ranges) from the cutoff color for each PCV range (Table 1).

## 4 Discussion

The current study confirms that visual inspection of hemolysis can be inaccurate, consistent with previous studies [13, 20]. Although visual inspection was performed without a color comparator in a previous veterinary study [13], a color scale was developed and used to aid visual inspection in this study. With the color scale, semiquantitative data regarding free hemoglobin concentration were provided, allowing not only for the determination of whether the survey participants made a correct color estimation but also for the distribution of incorrect estimations to be evaluated. It was found that all the incorrect estimations and potential incorrect transfusion decisions were observed when the sample supernatant color was one color range different (about 1 g/L of the free hemoglobin difference between adjacent colors on the scale) from the cutoff color. On the other hand, participants were able to differentiate the colors if the sample supernatant color was different by more than one color range from the cutoff color. The color survey results indicate that our hemolysis color scale can provide a level of guidance to clinicians when they evaluate the hemolysis level of canine RBC blood products. For example, when the degree of hemolysis of the RBC unit is more than one color range different from the cutoff color of the corresponding supernatant free hemoglobin concentration, an accurate estimation of the hemolysis level would be achieved by reading the color scale. When the degree of hemolysis of the unit is less than one color range different from the cutoff color, the differentiation of colors would start to be inaccurate, and the measurement of free hemoglobin would be necessary to calculate the exact percentage hemolysis. In other words, if the supernatant color of the unit is estimated to be near the cutoff color and within one color range, the free hemoglobin concentration of the unit should ideally be measured, and percentage hemolysis should be calculated to determine if it meets the <1% hemolysis standard.

In the current study, the percentage of potential correct decisions for the transfusion of the blood product as determined by the authors was 93.7% as opposed to 60% in the previous veterinary study [13]. This suggests that the color scale helped in the visual assessment of hemolysis. The percentage of correct decisions varied from 89.8% to 99.3% between the six different PCV ranges. It appears that wrong decisions occurred more often in the higher PCV ranges, which may suggest that the ability of the human eye to differentiate these colors is less accurate as the color gets darker or the level of hemolysis increases.

In this study, 29% of veterinarians, 33% of veterinary technicians and assistants, and 38% of veterinary students participated in the visual inspection of supernatant samples. Unlike the previous veterinary study [13] in which the participants were asked whether the degree of hemolyzed supernatant would be suitable to be transfused or not, the participants in the current study were asked to choose a color range in which the hemolyzed supernatant sample belonged. Although the level of experience with transfusions would not be expected to affect the study results, there could be differences between individuals in their ability to differentiate colors.

Both overestimation and underestimation of cell‐free hemoglobin can be problematic. Judging the hemoglobin concentration to be lower than it really is (underestimation) can lead to transfusion of a unit that has a higher percentage of hemolysis than is safe. On the other hand, judging the hemoglobin concentration to be higher than it is (overestimation) can lead to a unit being wasted that was actually acceptable.

During a pilot study, it was incidentally found the day after production of the supernatant samples that the color of the hematocrit tubes had faded while the author was cleaning the laboratory. Although it remains unclear why this phenomenon occurred, the supernatant being in the hematocrit tube for a long period of time might have altered the degree of oxygen binding of hemoglobin due to changes in its surroundings (e.g., oxygen concentration or penetration through the tube, changes in temperature). This finding should be further investigated in future studies.

The current study has some limitations. First, there was a small number of participants for the color survey. Because the supernatant color in the hematocrit tube changed over time during the initial pilot study, a decision was made to conduct the survey for only 2 h after production of the test samples. This limited the number of participants who could be enrolled into the study. Second, color changes may have occurred while the supernatant samples were photographed, when the photos were processed by the computer program, or when the color scale was printed. This color change may have affected the study results. To minimize the effect, the latest model of cell phone^e^ with a high specification built‐in camera was used, camera settings were standardized for all the photos taken, a professional computer graphics program was used by a professional graphic designer, and an optimal paper option was chosen based on colors of multiple printouts compared with the computerized colors on a laptop^i^ with a high specification of display. Third, while the hemoglobin analyzer in this study has been used in previous canine studies [13, 22, 23], it has not been validated for the accuracy of free hemoglobin measurement in veterinary patients. However, the measurements were reproducible and comparable to those of the gold standard tetramethylbenzidine method in human medicine [20]. Fourth, our color scale was expected to produce a degree of overestimation because the cutoff free hemoglobin value was assigned to each PCV group based on the low end of the PCV range to provide a safety margin and to avoid hemolysis >1%. Furthermore, participants in the visual inspection were not asked if they had any visual impairments that could affect the study results. However, no participant volunteered any concerns with color differentiation.

The use of <1% hemolysis as a standard is also open to discussion. While it is the standard for the FDA, European standards use <0.8% [24]. Both human and veterinary studies have demonstrated clinical complications from the administration of hemolyzed blood units; however, these studies have not measured the actual hemolysis levels. It may be that being one color “off” is still safe. Overestimation of the actual hemolysis and thus a decision not to use the unit could lead to excessive blood wastage.

In conclusion, the color scale developed in this study helped in the visual assessment of hemolysis, although visual inspection with the color scale can still be inaccurate near the corresponding cutoff color for 1% hemolysis in each PCV range. Correct color estimations or correct transfusion decisions were more likely to be made with test samples of lower hemoglobin concentration. For the area (less than one color range difference from the cutoff color) where the inaccuracy was noted, it is recommended to measure the free hemoglobin concentration and calculate the percentage hemolysis. Hemolysis color scales should continue to be validated in future studies using a larger number of samples. It may be desirable to incorporate technology (e.g., development of mobile applications) into the color scale analysis in order to improve the differentiation of colors.

## Author contributions

**Hyein Jung**: investigation, methodology, writing — original draft, writing — review and editing. **K. Jane Wardrop**: supervision, writing — review and editing. **Sabrina N. Hoehne**: supervision, writing — review and editing. **Linda G. Martin**: supervision, writing — review and editing. **Jillian M. Haines**: supervision, writing — review and editing. **Trey L. DeJong**: software, writing — review and editing. **Elizabeth B. Davidow**: supervision, writing — review and editing.

## Conflicts of Interest

The authors declare no conflicts of interest.

## Endnotes

a Fenwal quadruple blood‐pack pRBC units with Citrate Phosphate Dextrose Anticoagulant and Adsol red cell preservative solution, Washington State University Veterinary Teaching Hospital blood bank, Pullman, WA.

b Hemocue Plasma/Low Hb system, Hemocue America, Brea, CA.

c 0.9% Sodium Chloride Injection, USP, ICU Medical, Inc., Lake Forest, IL.

d Lux Light Meter Pro, Apple app store, Cupertino, CA.

e iPhone 14 Pro Max, Apple, Cupertino, CA.

f Built‐in camera application, Apple, Cupertino, CA.

g Adobe Illustrator, Adobe, San Jose, CA.

h Prism 9.0, Graph Pad Software, La Jolla, CA, USA; JASP Team (2023). JASP (Version 0.18.1) [Computer software]; IBM Corp. Released 2021. IBM SPSS Statistics for Windows, Version 28.0. Armonk, NY: IBM Corp.

i M1 Macbook Pro, Apple, Cupertino, CA.
